# Onjisaponin B Derived from *Radix Polygalae* Enhances Autophagy and Accelerates the Degradation of Mutant α-Synuclein and Huntingtin in PC-12 Cells

**DOI:** 10.3390/ijms141122618

**Published:** 2013-11-15

**Authors:** An-Guo Wu, Vincent Kam-Wai Wong, Su-Wei Xu, Wai-Kit Chan, Choi-In Ng, Liang Liu, Betty Yuen-Kwan Law

**Affiliations:** State Key Laboratory of Quality Research in Chinese Medicine, Macau University of Science and Technology, Macau, China; E-Mails: wag1114@foxmail.com (A.-G.W.); kawwong@must.edu.mo (V.K.-W.W.); suvia_xu@163.com (S.-W.X.); dk_kit@yahoo.com.hk (W.-K.C.); phoebeng112@gmail.com (C.-I.N.)

**Keywords:** autophagy, *Radix Polygalae*, onjisaponin B, AMPK, mTOR, α-synuclein, huntingtin, PC-12

## Abstract

Emerging evidence indicates important protective roles being played by autophagy in neurodegenerative disorders through clearance of aggregate-prone or mutant proteins. In the current study, we aimed to identify autophagy inducers from Chinese medicinal herbs as a potential neuroprotective agent that enhances the clearance of mutant huntingtin and α-synuclein in PC-12 cells. Through intensive screening using the green fluorescent protein-light chain 3 (GFP-LC3) autophagy detection platform, we found that the ethanol extracts of *Radix Polygalae* (Yuan Zhi) were capable of inducing autophagy. Further investigation showed that among three single components derived from *Radix Polygalae*—*i.e*., polygalacic acid, senegenin and onjisaponin B—onjisaponin B was able to induce autophagy and accelerate both the removal of mutant huntingtin and A53T α-synuclein, which are highly associated with Huntington disease and Parkinson disease, respectively. Our study further demonstrated that onjisaponin B induces autophagy via the AMPK-mTOR signaling pathway. Therefore, findings in the current study provide detailed insights into the protective mechanism of a novel autophagy inducer, which is valuable for further investigation as a new candidate agent for modulating neurodegenerative disorders through the reduction of toxicity and clearance of mutant proteins in the cellular level.

## 1. Introduction

Autophagy is a cellular process that involves the formation, expansion, and fusion of an isolated membrane to form double membrane vesicles (autophagosomes) that sequesters the cytoplasmic materials. Followed by fusion of the autophagosome with lysosome to form an autolysosome, all the engulfed materials are degraded by lysosomal hydrolases to recycle intracellular nutrients and energy [1,2]. Autophagy-lysosome and ubiquitin-proteasome pathways are the two major protein degradation pathways in cells [3]. While short-lived nuclear and cytosolic proteins are degraded by proteasomes, large membrane proteins, oligomers and aggregates which cannot pass through the narrow pore of the proteasome barrel are degraded by autophagy [4].

A variety of neurodegenerative diseases are caused by toxic, aggregate-prone or oligomeric proteins [5–9]. For example, Huntington disease (HD) is caused by an over 35 CAG trinucleotide repeat expansion, which results in a long mutant polyQ tract in the huntingtin protein. These polyQ expansions are highly associated with the aggregate formation and toxicity [10,11]. Autophagy, however, can reduce mutant huntingtin levels and its toxicity in cell and mouse models [6,7]. Parkinson disease (PD) is caused by A53T or A30P α-synuclein mutants, which are identified as substrates of autophagy, and the clearance of these mutant proteins are also highly dependent on autophagy [6–8,11–14]. Pharmacological activation of autophagy reduces the levels and toxicity of mutant huntingtin, mutant proteins in spinocerebellar ataxia, mutant α-synuclein and mutant tau in either mouse or drosophila models [5,15]. Furthermore, autophagy-related gene (*Atg* knockdown leads to aggregate formation and toxicity in *C. elegans* [16,17]. In addition to protein aggregate formation, the accumulation of abnormal mitochondria or endoplasmic reticulum, and an increase in the size and number of lipid droplets were observed in *Atg* knockout animal models [14,18–20].

Chinese medicinal herbs have been used in treating neurodegenerative diseases such as dementia or Alzheimer’s disease for years. Recently, researchers have isolated many novel compounds from medicinal herbs, of which, some were found to be effective in modulating neurodegenerative disorders [21]. For example, salidroside can protect against amyloid-β peptide (Aβ)-induced oxidative damage on PC-12 cells and SH-SY5Y neuroblastoma cells [22]. Besides, it also protects against streptozotocin-induced neural injury in rat [23]. Curcumin has been demonstrated to be an effective agent against amyloid β-aggregation [24–26] which is responsible for the pathogenesis of Alzheimer’s disease [27]. Huperzine A, an alkaloid extracted from the plant *Huperzia Serrate*, is widely used in the mainland China to treat Alzheimer’s disease [28] and was reported to reduce glutamate-induced toxicity in neurons [29]. Therefore, identification of new neuroprotective compounds from Chinese medicinal herbs becomes an important part of novel drug discovery.

Recent literatures have described new chemicals that enhance the autophagic clearance of mutant proteins [30]. For instance, rapamycin, an inducer of mammalian target of rapamycin (mTOR)-dependent autophagy, increases autophagic clearance of mutant huntingtin fragments *in vivo* [5,7,31]. However, mTOR inhibition has adverse effects in protein synthesis, cell proliferation and immune function [1,32]. Therefore, drugs that can enhance the autophagic clearance of mutant or aggregate-prone proteins with minimal side effects would be highly desirable.

In this study, we aimed to identify novel autophagy inducers with neuroprotective effects by evaluating their efficacy in reducing the protein level and toxicity of mutant huntingtin and A53T α-synuclein. Through screening of our library of natural product extracts from Chinese medicinal herbs, it was demonstrated that both *Radix Polygalae* and its single component, onjisaponin B, are able to induce autophagy with potential therapeutic application in modulating neurodegeneration. With a long history of use in traditional Chinese medicine to relieve insomnia, anxiety and heart palpitation [33], *Radix Polygalae* and its active components were proved to be capable of improving learning and memory of aged mice [34,35], attenuating hepatic ischemia-reperfusion induced cognitive dysfunction [36], inhibiting toxin-induced neuronal death in the Parkinson’s disease models [37], as well as having cerebral protective and cognition-improving effects [38]. Therefore, the study of the neuro-protective mechanisms and therapeutic applications of *Radix Polygalae* and its active components would be important for novel drug discovery.

## 2. Results and Discussion

### 2.1. The Ethanol Extracts of Radix Polygalae Induce Autophagy in PC-12 Cells

**Figure 1:**
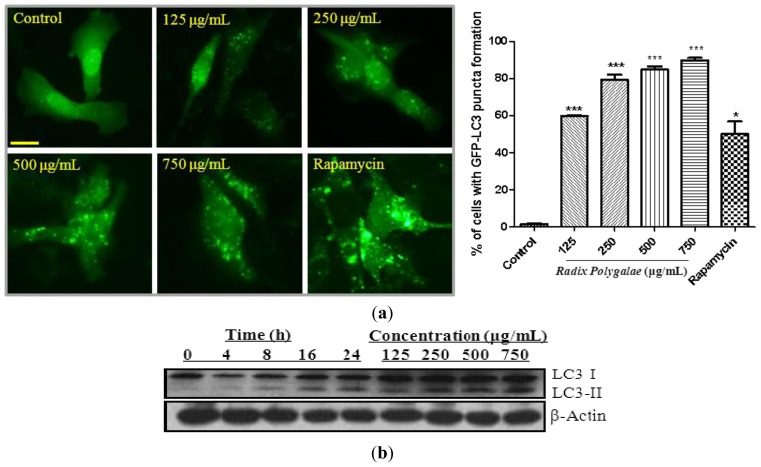
*Radix Polygalae* ethanol extracts induce autophagy. (**a**) Representative images showing the formation of GFP-LC3 puncta in PC-12 cells after treatment of *Radix Polygalae* ethanol extracts at the indicated concentrations for 24 h. Rapymycin (0.3 μM) was used as a positive control. Right: bar chart represents the percentage of cells with GFP-LC3 puncta formation. Magnification, ×40; (**b**) PC-12 cells were treated with *Radix Polygalae* ethanol extract for the indicated time and concentrations. Cell lysates were analyzed by western blot for LC3 conversion (LC3-I, 18 kDa; LC3-II, 16 kDa) and β-actin, respectively. ***p < 0.001; *p < 0.05. Scale bar: 15 μm.

According to Shenong’s report in the Liang Dynasty of China, Chinese medicinal herbs which are commonly prescribed for tranquilization and relieving convulsion and spasm were selected for the study. We monitored autophagy induction by detecting the conversion of cytosolic LC3-I to membrane-bound LC3-II using immunoblotting and immunofluorescence microscopy [39,40]. Immunofluorescence detection was performed by transiently expressing PC-12 cells with GFP-LC3 [41], and then cells were incubated with different Chinese herbal ethanol extracts, respectively. By quantitating the percentage of cells with GFP-LC3 puncta formation, we found that the partially purified ethanol extract from *Radix Polygalae* (Yuan Zhi), a medicinal herb commonly used to mediate depression [42,43], increased the percentage of cells with GFP-LC3 puncta formation (Figure 1a) in a dose dependent manner. To further confirm its autophagic activity, PC-12 cells were treated with *Radix Polygalae* ethanol extract for different durations and concentrations. Cells were then analyzed by western blot for LC3-I to LC3-II conversion, which is a crucial step for autophagy induction [39,44,45]. Our immunoblotting results showed that *Radix Polygalae* extract significantly increased the conversion of LC3-I to LC3-II in a time- and dose-dependent manner (Figure 1b).

### 2.2. The Ethanol Extracts of Radix Polygalae Induce Autophagy through an *Atg*7 Dependent Manner

**Figure 2:**
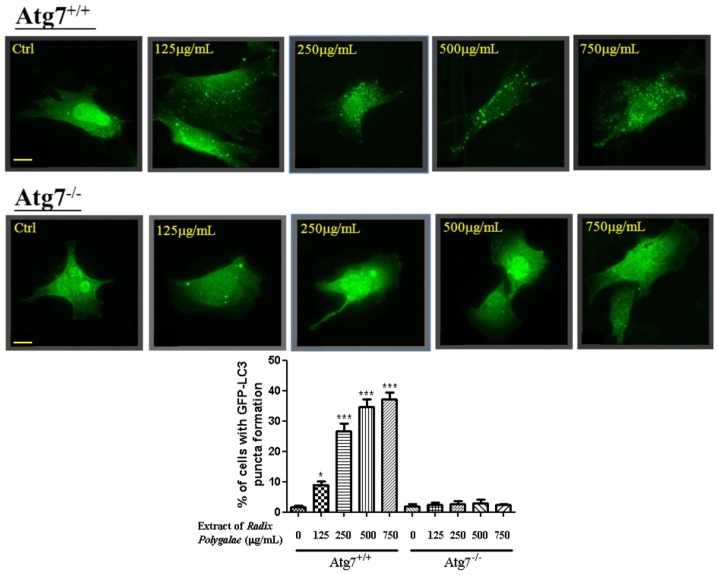
Ethanol extracts of *Radix Polygalae* induce autophagy via an *Atg*7 dependent mechanism. Wild type *Atg*7 and *Atg*7-deficient MEF cells transiently transfected with GFP-LC3 were treated with *Radix Polygalae* ethanol extracts at the indicated concentrations for 24 h. Bottom: bar chart represents the percentage of cells with GFP-LC3 puncta formation. Magnification, ×40. ***p < 0.001; *p < 0.05. Scale bar: 15 μm.

To further examine the effect of *Radix Polygalae* ethanol extract on autophagy induction, wild type autophagy gene 7 (*Atg*7) and *Atg*7-deficient mouse embryonic fibroblasts (MEFs) were employed for the study. Upon autophagy induction, LC3-I is activated by *Atg*7 and transferred to the *Atg*3. Then, LC3-I is conjugated to phosphatidylethanolamine (PE) [46] and become membrane bound LC-3-II which leads to autophagosome formation [47]. As demonstrated by immunofluorescence microscopy, *Radix Polygalae* ethanol extract induced GFP-LC3 puncta formation in wild type *Atg*7 cells but not in *Atg*7-deficient mouse embryonic fibroblast, which is resistant to autophagy induction (Figure 2) [48]. These data suggested that *Radix Polygalae* ethanol extract work as a potent autophagy enhancer in cellular model and its autophagy effect is dependent on *Atg*7 gene regulation.

### 2.3. The Ethanol Extracts of Radix Polygalae Inhibit the mTOR Signaling Pathway

**Figure 3:**
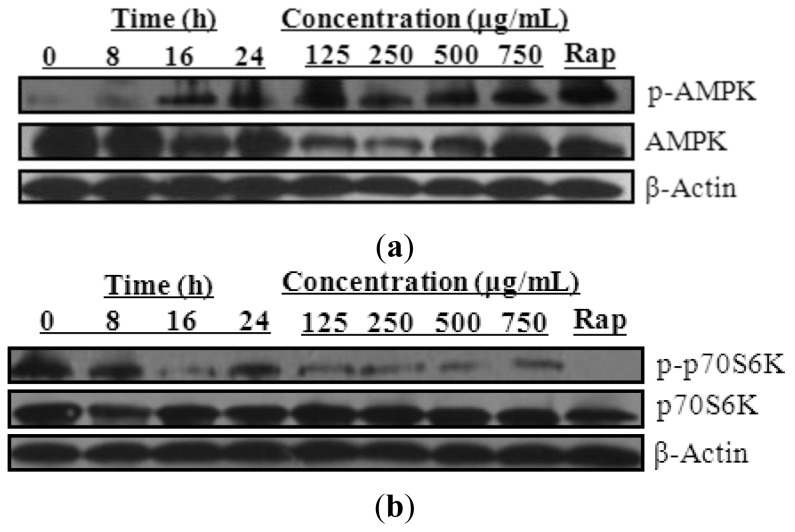
*Radix Polygalae* ethanol extracts activate the AMPK-mTOR signaling pathway. (**a**) PC-12 cells were treated with *Radix Polygalae* ethanol extract at the indicated time and concentrations, respectively. Cells treated with 0.3 μM of rapamycin (Rap) for 24 h were used as the positive control. Cell lysate was then harvested and analyzed for p-AMPK and AMPK; or (**b**) p-p70S6K and p70S6K, respectively.

Extensive studies showed that autophagy is activated through inhibition of the mammalian target of rapamycin (mTOR), a central cell-growth regulator that integrates growth factor and nutrient signals [49,50]. The key energy sensor—AMP activated protein kinase (AMPK)—in turn, can inhibit mTOR through TSC2 phosphorylation [51]. As shown in Figure 3a, PC-12 cells treated with *Radix Polygalae* ethanol extract showed an increase in AMPK phosphorylation in a time- and dose-dependent manner. This was accompanied by a concomitant reduction in phosphorylated p70S6K (Figure 3b), which acts as the downstream target of mTOR. Taken together, these data suggested that *Radix Polygalae* ethanol extracts are able to induce autophagy through the AMPK-mTOR pathway.

### 2.4. Identification of Onjisaponin B as a Novel Autophagy Inducer

**Figure 4:**
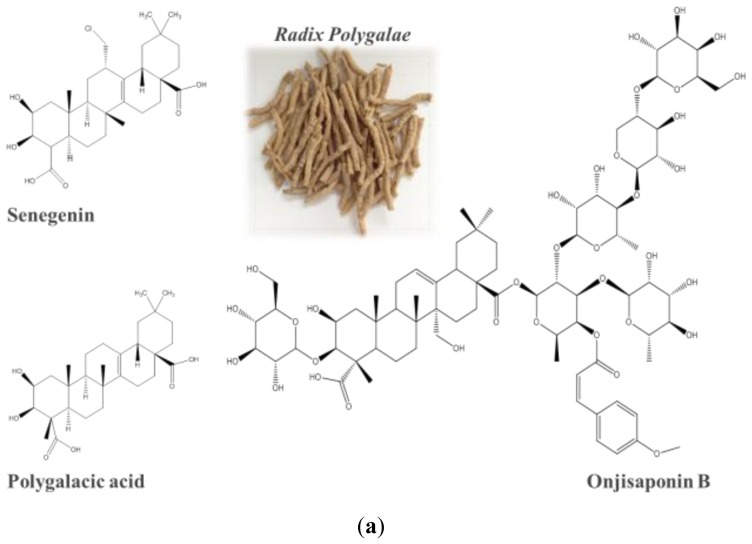

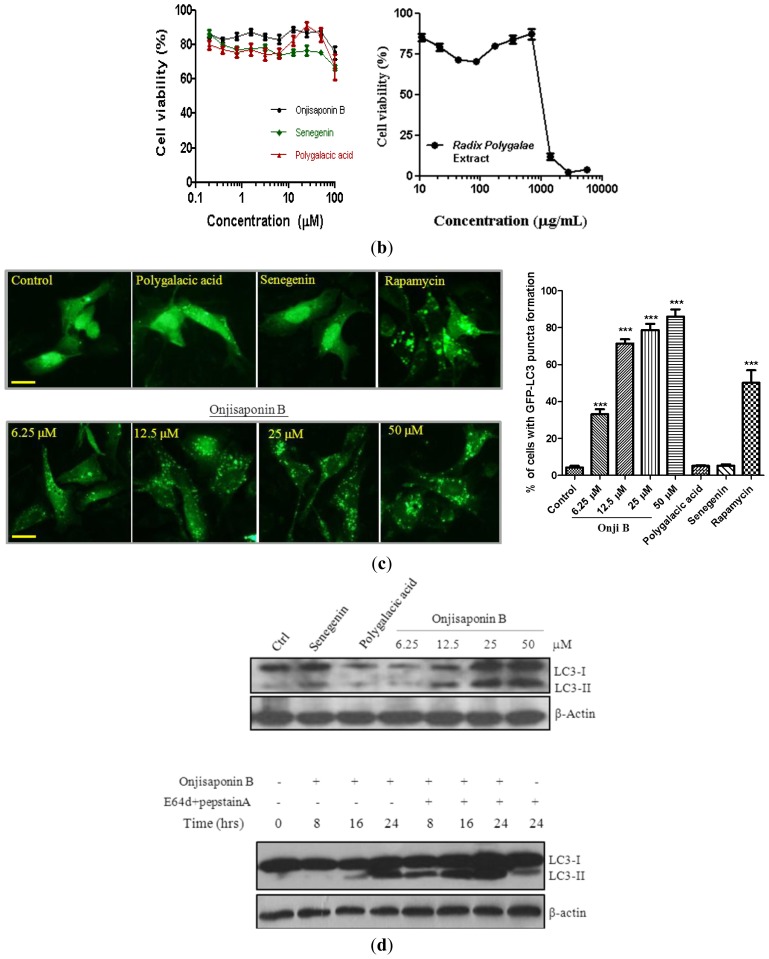

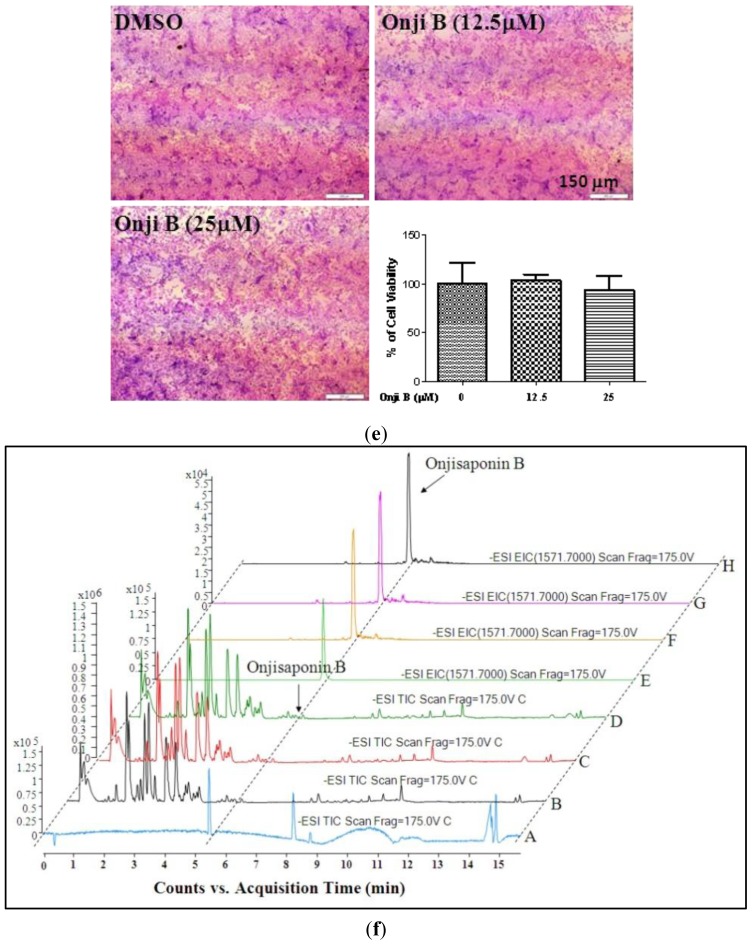
Onjisaponin B as a novel autophagy inducer. (**a**) Chemical structure of polygalacic acid, senegenin and onjisaponin B; (**b**) Cytotoxicity of polygalacic acid, senegenin, onjisaponin B and *Radix Polygalae* crude extract against PC-12 cells after 48 h of drug treatment; (**c**) PC-12 cells transfected with GFP-LC3 plasmids were incubated with polygalacic acid (50 μM), senegenin (50 μM), rapaymcin (0.3 μM) and 0–50 μM of onjisaponin B (Onji B) for 24 h. **Left**: representative images with GFP-LC3 puncta formation (magnification, ×40); **right**: bar chart indicated the percentage of cells with GFP-LC3 puncta formation under these treatments; bars, S.D. ***p < 0.001; (**d**) PC-12 cells were treated with polygalacic acid (50 μM), senegenin (50 μM) and 0–50 μM of onjisaponin B for 24 h. **Lower**: onjisaponin B (25 μM) was treated with the presence of lysosomal protease inhibitors (10 μg/mL) for the indicated time. Cell lysates were then harvested and analyzed for LC3 I/II and β-actin, respectively; (**e**) PC-12 cells were stained with crystal violet for cell visualization after 24 h of onjisaponin B treatment at the indicated concentrations. Bright field images were captured (magnification, ×4). Columns, means of three independent experiments; (**f**) The mass spectrum of onjisaponin B and *Radix Polygalae* ethanol extract. All samples were analyzed by UHPLC-MS/TOF and separated on an Agilent Zorbax Eclipse Plus C-18 (50 mm × 2.1 mm) column (particle size: 1.8 μm) at a flow rate of 0.35 mL min^−1^. The data was acquired in the scan mode from *m*/*z* 100–1700 Da with 2.0 spectra/s. (**A**): The Total Ion Chromatogram (TIC) of onjisaponin B in the standard solution; (**B**–**D**): The TIC of three different batch of *Radix Polygalae* ethanol extracts; (**E**): The Extracted Ion Chromatogram (EIC) of onjisaponin B in the standard solution; (**F**–**H**): The EIC of onjisaponin B in the corresponding TIC (**B**–**D**) of three different batch of *Radix Polygalae* ethanol extracts. Scale bar: 15 μm.

To identify the active components responsible for the autophagy activity of the ethanol extracts of *Radix Polygalae*, three single compounds isolated from *Radix Polygalae* including polygalacic acid, senegenin and onjisaponin B (Figure 4a) were further investigated. There was no overt toxicity observed in PC-12 cells treated with polygalacic acid, senegenin or onjisaponin B for 48 h as revealed by MTT assay (Figure 4b). Furthermore, while polygalacic acid and senegenin showed no autophagy activity in cells (Figure 4c), onjisaponin B increased the formation of GFP-LC3 puncta formation in a dose-dependent manner as revealed by fluorescent microscopy (Figure 4c). Furthermore, it increased the rate of LC3-II formation with the presence of protease inhibitors as revealed by western blot analysis (Figure 4d) and with no obvious cytotoxicity observed (Figure 4e).

Further analysis of the ethanol extracts of *Radix Polygalae* using UHPLC-MS/TOF was performed in the scan mode from *m*/*z* 100–1700 Da with 2.0 spectra/s. In the range of the accurate mass from 100–1700 Da, it was found that 90 out of 91 compounds presented in the ethanol extracts of *Radix Polygalae*, matched with the compounds found in its herbal plant *Polygalae Tenuifolia* (Figure 4f) according to the Dictionary of Natural Products [52]. Furthermore, we confirmed that the concentration of onjisaponin B presented in the ethanol extract of *Radix Polygalae* was 4.28 μM as revealed by the standard curve deduction (data not shown). Taken together, these data indicated that onjisaponin B is probably the most active component responsible for the autophagy effect of *Radix Polygalae* extract. Besides, with no obvious cytotoxicity observed after treatments, onjisaponin B may work as a potential therapeutic agent for modulating neurodegenerative disorders in the future.

### 2.5. Onjisaponin B Activates the AMPK-mTOR Signaling Pathway

**Figure 5:**
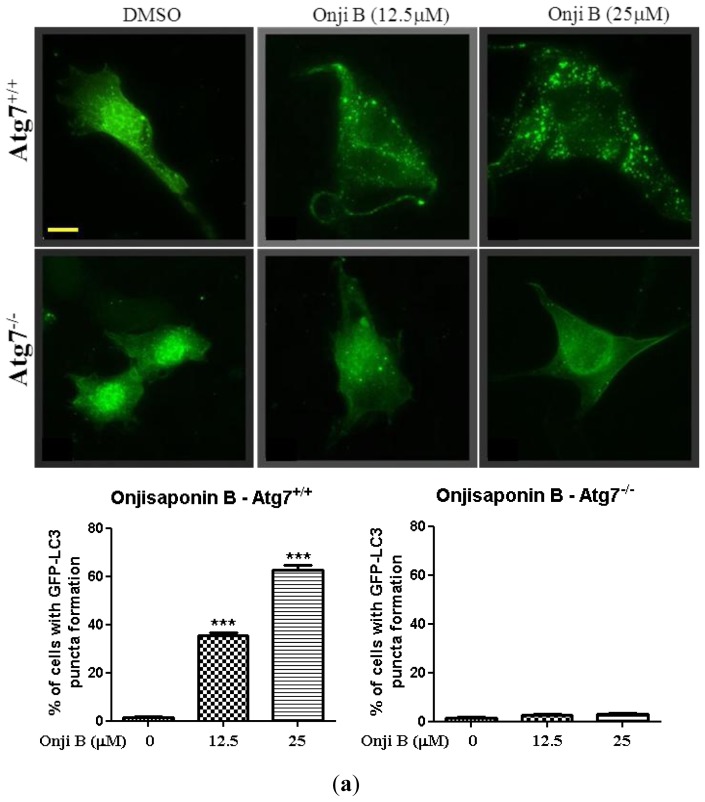

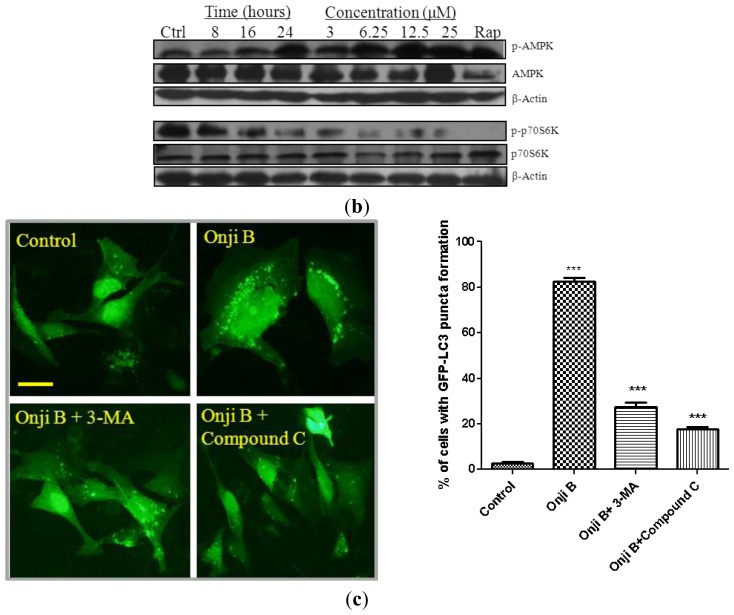
Onjisaponin B activates autophagy through an *Atg*7, AMPK-mTOR dependent pathway. (**a**) Wild type *Atg*7 and *Atg*7-deficient MEFs transfected with GFP-LC3 were treated with onjisaponin B (Onji B) at the indicated concentrations for 24 h. **Bottom**: bar chart indicated the percentage of cells with GFP-LC3 puncta formation under these treatments; (**b**) PC-12 cells were treated with onjisaponin B at the indicated time and concentrations for 24 h. Cells treated with 0.3 μM of rapymycin (Rap) was used as a positive control. Cell lysate was then harvested and analyzed for p-AMPK, AMPK, p-p70S6K and p70S6K, respectively; (**c**) PC-12 cells expressing GFP-LC3 were treated with onjisaponin B (25 μM) in the presence of compound C (CC, 5 μM) or 3-Methyladenine (3-MA, 5 mM) for 12 h. Representative images with punctate GFP-LC3 fluorescence were captured. Bar chart indicated the percentage of cells with GFP-LC3 puncta formation under these treatments. Magnification, ×40. Columns: means of three independent experiments. Bars, S.D. ***p < 0.001. Scale bar: 15 μm.

To further investigate the molecular mechanism of onjisaponin *B*, it was shown that onjisaponin B induced GFP-LC3 puncta formation in wild type *Atg*7 cells but not in *Atg*7-knockout mouse embryonic fibroblasts (Figure 5a), which are resistant to autophagy induction. This result suggests that onjisaponin B work as a novel autophagy enhancer which depends on autophagy related gene *Atg*7 for autophagy induction.

Furthermore, onjisaponin B activated the phosphorylation of AMPK in a time- and dose-dependent manner (Figure 5b), and this activation was also accompanied by a concomitant reduction in its downstream p70S6K phosphorylation (Figure 5b). In addition, there was a significant reduction in GFP-LC3 puncta formation in PC-12 cells treated with the presence of AMPK inhibitor (compound C) or the autophagy inhibitor (3-Methyladenine, 3-MA) (Figure 5c), which is a specific inhibitor of the class III PI3K responsible for autophagy induction [53]. Collectively, these data suggested that with a similar molecular mechanism, onjisaponin B activates autophagy through an *Atg*7, AMPK-mTOR dependent manner.

### 2.6. Onjisaponin B Enhances the Clearance of Mutant Huntingtin and A53T α-Synuclein and Reduces Oligomerization of α-Synuclein

Huntington disease (HD) is a neurodegenerative disease characterized by CAG trinucleotide repeat expansion which results in mutant huntingtin formation [11]. On the other hand, another well-known neurodegenerative condition, Parkinson disease (PD), is associated with the A53T mutant aggregated protein formation [54,55]. The clearance of these two autophagy substrates is highly dependent on autophagy [6–8,12,13]. As a potent autophagy inducer isolated from *Radix Polygalae*—a Chinese medicinal herb that is commonly prescribed for neurodegenerative diseases—we therefore postulated that onjisaponin B is able to enhance the clearance of mutant huntingtin and α-synuclein *in vitro*.

**Figure 6:**
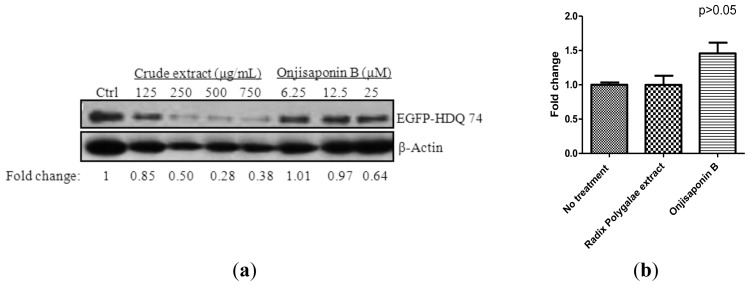

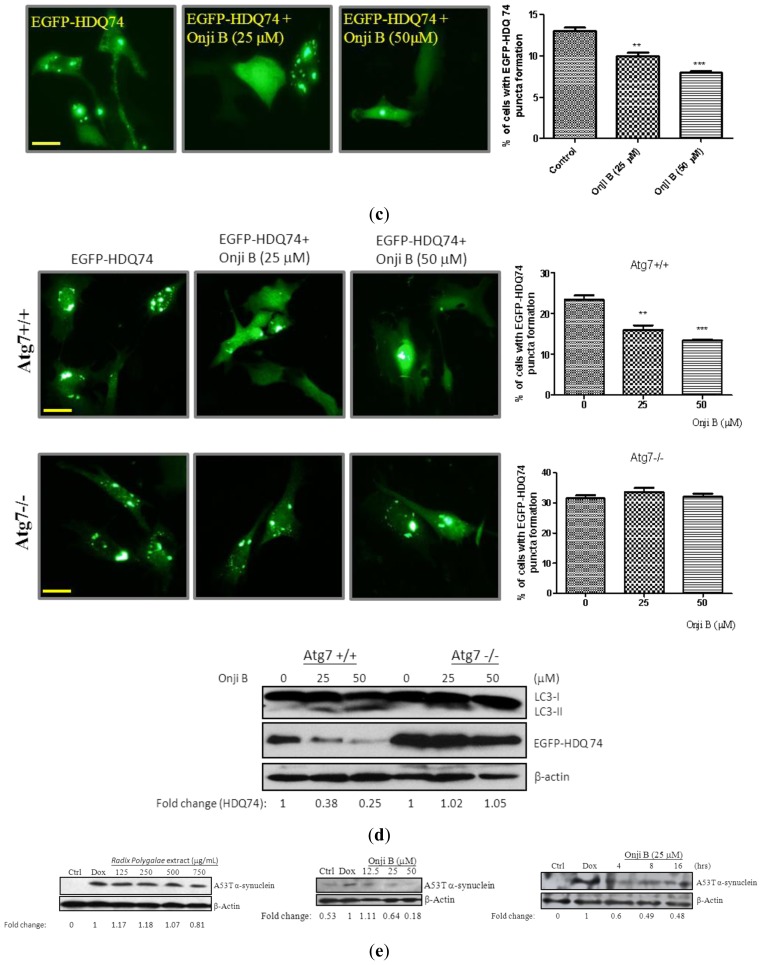

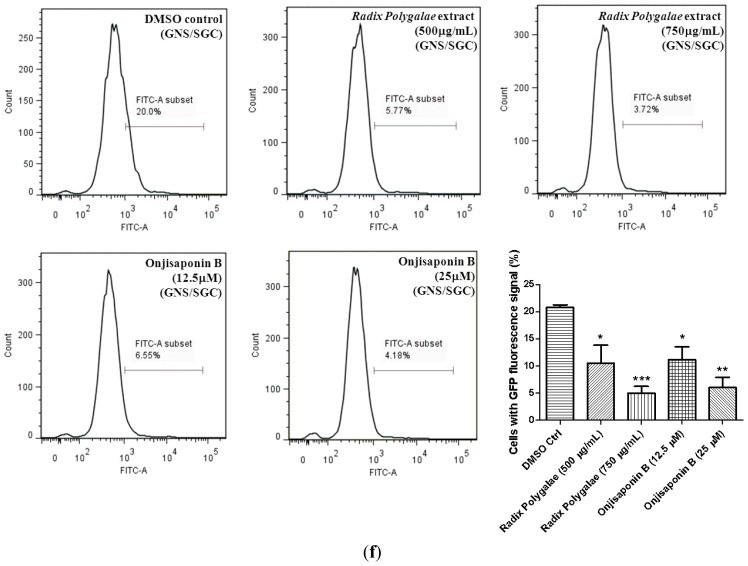
*Radix Polygalae* ethanol extract and onjisaponin B accelerate the clearance of mutant huntingtin and A53T α-synuclein, and inhibits α-synuclein oligomerization. (**a**) PC-12 cells transfected with huntingtin with 74 CAG repeats were treated with 24 h of *Radix Polygalae* extract or onjisaponin B at the indicated concentrations. Cell lysate was harvested and analyzed for EGFP and β-actin respectively; (**b**) PC-12 cells transfected with EGFP-HDQ 74 were analyzed by real time PCR on huntingtin level after 16 h of *Radix Polygalae* extract (500 μg/mL) or onjisaponin B (50 μM) treatment; (**c**) PC-12 cells transfected with EGFP-HDQ 74 were incubated with onjisaponin B (25 and 50 μM) for 24 h before fluorescent microscopy analysis; (**d**) Wild type *Atg*7 and *Atg*7-deficient MEF cells transiently transfected with EGFP-HDQ 74 were treated with 0–50 μM of onjisaponin B for 24 h before fluorescent microscopy analysis. Bar chart represents the percentage of cells with EGFP-HDQ 74 inclusion formation. Magnification, ×40. **Bottom**: Wild type *Atg*7 and *Atg*7-deficient MEF cells with EGFP-HDQ 74 overexpression were treated with 0–50 μM of onjisaponin B for 24 h before western blot analysis. Cell lysate was harvested and analyzed for EGFP, LC3 and β-actin respectively; (**e**) Doxycycline-inducible PC-12 cell lines transfected with mutant A53T α-synuclein were first induced with doxycycline (1 μg/mL) for 24 h and the expression of transgene was then switched off by the removal of doxycycline. Cells were then treated with *Radix Polygalae* extract (**left**) or onjisaponin B (**middle**) at the indicated concentrations for a further 24 h after doxycycline removal. Ctrl: cells without the addition of doxycycline; Dox: cells with the removal of doxycycline (1 μg/mL) after 24 h of induction but without *Radix Polygalae* extract or onjisaponin B treatment. **Right**: doxycycline-inducible PC-12 cell lines transfected with mutant A53T α-synuclein were incubated with onjisaponin B (25 μM) at the indicated time points. Band intensities of the blot were quantified using densitometric analysis and normalized to β-actin. Data is expressed as a fold change over control; (**f**) HeLa cells transfected with both GNS and SGC plasmids were incubated with *Radix Polygalae* extract or onjisaponin B at the indicated concentrations. **Right**: bar chart indicated the percentage of cells with green fluorescent protein (GFP)-positive signal under these treatments. Data from the flow cytometry analysis is represented as means ± S.D. of three independent experiments. ***p < 0.001; **p < 0.01; *p < 0.05. Scale bar: 15 μm.

To this end, we transiently overexpressed the mutant huntingtin with 74 CAG trinucleotide repeats (EGFP-HDQ 74) in PC-12 cell line. As shown in Figure 6a, both ethanol extract of *Radix Polygalae* and onjisaponin B enhanced the clearance of overexpressed EGFP-tagged mutant huntingtin with 74 CAG repeats as measured by immunoblotting against GFP antibody. Real time PCR analysis was performed to ensure that both ethanol extract of *Radix Polygalae* and onjisaponin B did not affect the transcriptional level of huntingtin significantly (Figure 6b). Immunocytochemistry analysis further confirmed that onjisaponin B enhanced the clearance of inclusions formed by EGFP-HDQ 74 (Figure 6c). To further confirm the protective effect of onjisaponin B was due to an *Atg*7 dependent pathway, wild type *Atg*7 cells and *Atg*7-knockout mouse embryonic fibroblasts were transfected with EGFP-HDQ 74 for fluorescent inclusions formation. Our results showed that onjisaponin B enhanced the rate of LC3II formation and the clearance of EGFP-HDQ 74 inclusions in wild type *Atg*7 cells but not in *Atg*7-knockout cells (Figure 6d), suggesting the effect was autophagy dependent.

Next, we examined if onjisaponin B enhances the clearance of another autophagy substrate, mutant A53T α-synuclein, by using a doxycycline-inducible PC-12 cell line. In this cellular model, the expression of A53T α-synuclein can be switched on by adding the chemical agent, doxycycline. Upon the induction by doxycycline, we can evaluate whether specific compounds enhance the clearance of the induced mutant protein [11,30,56]. As shown in Figure 6e, both *Radix Polygalae* ethanol extract and onjisaponin B accelerate the clearance of myc-tagged mutant A53T α-synuclein, whereas cells without onjisaponin B or ethanol extract incubation showed no removal of mutant protein after doxycycline induction. Collectively, these data suggested that both ethanol extract of *Radix Polygalae* and onjisaponin B may work as a useful neuroprotective agent through accelerating the clearance of mutant huntingtin and α-synuclein *in vitro*, thus, further investigation would need to be done.

Other than the mutations in α-synuclein [54,57], misfolding, fibrillization or oligomerization of α-synuclein also play an important role in the pathogenesis of Parkinson’s disease (PD) [58,59]. Here, by using the bimolecular fluorescence complementation (BiFC) assay, we are able to directly quantify the oligomerization of α-synuclein in living cells [59]. In brief, α-synuclein proteins fused with two different non-fluorescent GFP terminal fragments, (i) GFP-N terminal-α-synuclein (GNS) or (ii) α-synuclein-GFP-*C* terminal (SGC), respectively, were used [59,60]. Upon oligomerization of α-synuclein, the two non-fluorescent fractions of GFP will therefore reconstitute the complete GFP fluorophore and give out GFP fluorescent signal within cells. This signal in turn can be quantified by flow cytometry analysis. As measured by flow analysis in Figure 6f, both *Radix Polygalae* crude extract and onjisaponin B inhibit the oligomerization of α-synuclein in HeLa cells transfected with GNS and SGC. HeLa cells were selected for the BiFC study due to their high transfection efficiency [60,61]. The decrease of GFP fluorescent signal suggested that both *Radix Polygalae* ethanol extract and its active component onjisaponin B may play a protective role in Parkinson’s disease through inhibiting α-synuclein oligomerization, which is a crucial step for α-synuclein aggregation. Taken together, these novel findings provide evidence to further support the neuroprotective function of *Radix Polygalae* ethanol extract and onjisaponin B in cellular model.

### 2.7. Onjisaponin B Has Protective Effect on the Toxicity of Mutant Huntingtin and A53T α-Synuclein Proteins

**Figure 7:**
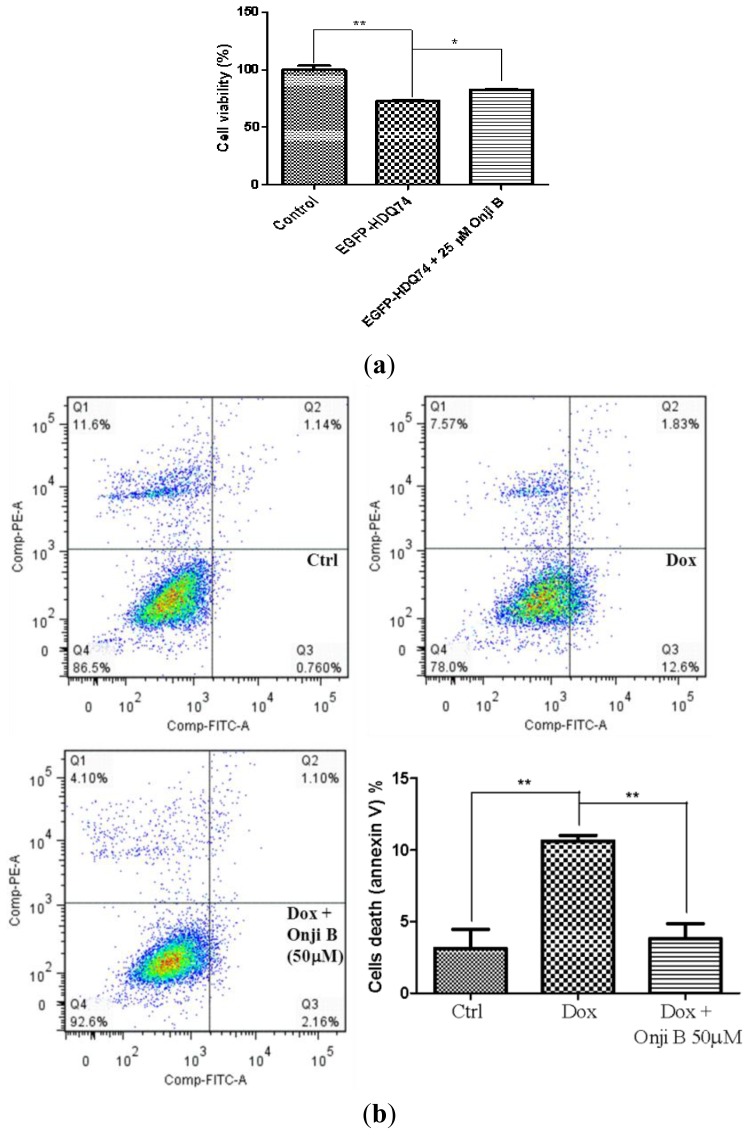
Onjisaponin B reduces toxicity in PC-12 cells expressing either mutant huntingtin or A53T α-synuclein. (**a**) PC-12 cells transfected with huntingtin with 74 CAG repeats for 12 h were treated with 48 h of onjisaponin B. Cell death was assessed by MTT assay; (**b**) Doxycycline-inducible PC-12 cell lines transfected with mutant A53T α-synuclein were induced with doxycycline (1 μg/mL) for 24 h and the expression of transgene was then switched off by the removal of doxycycline. Cells were then treated with onjisaponin B (50 μM) for a further 24 h after doxycycline removal. Ctrl: cells without the addition of doxycycline; Dox: cells with the removal of doxycycline (1 μg/mL) after 24 h of induction but without onjisaponin B treatment. Cell death was then assessed by flow cytometry. Data from the bar chart represented the means ± S.D. of three independent experiments. **p < 0.01; *p < 0.05.

Many studies have demonstrated that overexpression of mutant proteins in cultured cell models are highly associated with cellular toxicity [30,62–64]. Therefore, with the result that onjisaponin B treatment enhanced the clearance of mutant aggregate-prone proteins, we next investigated if onjisaponin B plays a protective role in reducing cell death induced by mutant huntingtin or A53T α-synuclein expression. To study the toxicity of mutant huntingtin or A53T α-synuclein in cells, PC-12 cells transfected transiently with EGFP-HDQ 74 or myc-tagged mutant A53T α-synuclein were used to examine the effect of onjisaponin B on cell viability. As shown in Figure 7, while transient expression of mutant huntingtin or A53T α-synuclein lead to a decrease in cell viability, the addition of onjisaponin B reduced toxicity in PC-12 cells expressing either mutant huntingtin (Figure 7a) or A53T α-synuclein (Figure 7b), respectively. Consistent with our previous findings that onjisaponin B increases the clearance of mutant huntingtin and A53T α-synuclein and reduces oligomerization of α-synuclein, our results further demonstrated the potential therapeutic role of onjisaponin B working as a neuroprotective agent, through lowering of mutant huntingtin and α-synuclein toxicity in cells.

## 3. Experimental Section

### 3.1. Reagents, Chemicals, Antibodies and Plasmids

All chemicals and reagents were purchased from Sigma-Aldrich (St. Louis, MO, USA) unless otherwise specified. Compound C was obtained from Calbiochem (Frankfurter Straβe, Darmstadt, Germany). Polygalacic acid, senegenin and onjisaponin B (>98% purity, HPLC) were purchased from Chengdu MUST Bio-technology Company Ltd. (Chengdu, China). pEGFP-LC3 reporter plasmid was a generous gift from Tamotsu Yoshimori (Osaka University, Osaka, Japan). EGFP-HDQ 74 plasmid was kindly provided by David C. Rubinsztein (University of Cambridge, Cambridge, UK). Antibodies against LC3B, p-AMPK (Thr172), AMPK, p-p70S6K (Thr389), p70S6K and myc-tag were purchased from Cell Signaling Technologies Inc. (Beverly, MA, USA). GFP antibodies were purchased from Santa Cruz Biotechnology (Santa Cruz, CA, USA). β-actin antibodies were from Sigma (St. Louis, MO, USA).

### 3.2. Preparation of the Standardized Ethanol Extracts of Radix Polygalae

Standardized ethanol extract of *Radix Polygalae* were used throughout the experiments. In brief, *Radix Polygalae* was purchased from the Luo Shen Xing Zhong Yao Cai Co. Ltd. (Hebei, China). The quality of *Radix Polygalae* conforms to the requirement of Hong Kong Standard of Chinese Materia Medica. Appropriately 20 g of *Radix Polygalae* were smashed into powder and then extracted with 10 times its volume of 75% ethanol for 1 h by using the refluxing extraction method. The extraction was repeated two times. The extracted solution was then filtered, concentrated, and dried with a rotatory evaporator under reduced pressure to give the final herbal ethanol extracts. The ethanol extracts were weighed and then dissolved in DMSO at a suitable concentration until further use.

UHPLC analysis was performed by Agilent Technologies 1290 Series UHPLC (Agilent Technologies, Santa Clara, CA, USA) equipped with an Agilent Technologies 6230 Time of Flight MS (Agilent Technologies, Santa Clara, CA, USA) equipped with a Jet Stream ion source (Agilent Technologies, Santa Clara, CA, USA) operated in negative ion mode. The samples were analyzed on an Agilent Zorbax Eclipse Plus C-18 (50 mm × 2.1 mm) column (particle size: 1.8 μm); Agilent Technologies, Santa Clara, CA, USA) at a flow rate of 0.35 mL min^−1^. The column temperature was 40 °C and the injection volume was 1 μL. A gradient elution program was applied with mobile phase A: 0.1% formic acid in water and mobile phase B: 0.1% formic acid in ACN as follows: 0–8 min, 5%–70% (B); 8–11 min, 70%–100% (B); 11–14 min, 100% (B); 14–18 min, 5% (B). In case of LC-MS/TOF, two reference masses (*m*/*z* 112.98558700 and 119.03632000) were used, respectively. The most relevant MS parameters were set as follow: fragmentor voltage: 175 V; capillary voltage: 4000 V; nebulizer pressure: 40 psi; gas temperature: 325 °C; gas flow rate: 11 L min^−1^. The data was acquired in the scan mode from *m*/*z* 100 to 1700 Da with 2.0 spectra/s. Data analysis was carried out using Agilent MassHunter Workstation software B.01.03 (Agilent Technologies, Santa Clara, CA, USA).

### 3.3. Cell Culture

All cells were obtained from the American Type Culture Collection (ATCC) (Rockville, MD, USA) unless otherwise stated. Wild type *Atg*7 and *Atg*7-deficient mouse embryonic fibroblasts (MEF) were generous gifts from Masaaki Komatsu (Juntendo University, School of Medicine, Tokyo, Japan). All cells were cultured with medium supplemented with 10% fetal bovine serum, 50 U/mL penicillin and 50 μg/mL streptomycin (Invitrogen, Scotland, UK) in a 5% humidified CO_2_ incubator at 37 °C, except PC-12 cells which were supplemented with 10% horse serum. Doxycycline inducible PC-12 cell line was maintained in DMEM supplemented with 10% horse serum, 5% of Tet system approved fetal bovine serum (Clontech, Mountain View, CA, USA) and 200 μg/mL of G418 in the presence of 10% CO_2_.

### 3.4. Quantification of GFP-LC3 Puncta Formation

GFP-LC3 puncta formation was quantified as described previously [2]. In brief, cells grown on coverslips in a 6-well plate were fixed in 4% paraformaldehyde for 20 min at room temperature and then rinsed with PBS. Slides were mounted with FluorSave™ mounting media (Calbiochem, San Diego, CA, USA) and examined by fluorescence microscopy. The number of GFP-positive cells with GFP-LC3 puncta formation was examined under the Nikon ECLIPSE 80i microscope. Representative images were captured with CCD digital camera Spot RT3™ (Diagnostic Instruments, Inc., Melville, NY, USA). To quantify autophagy, the percentage of cells with punctate GFP-LC3 fluorescence was calculated by counting the number of the cells with punctate GFP-LC3 fluorescence in GFP-positive cells. A minimum of 150 cells from 3 randomly selected fields were scored.

### 3.5. Cytotoxicity Assays

All compounds were dissolved in DMSO and stored at −20 °C until further use. Cell viability was measured by using the MTT method (3-[4,5-dimethylthiazol-2-yl]-2,5-diphenyl tetrazolium bromide) as described previously [65]. For cell viability assay measured by crystal violet staining, PC-12 cells were incubated in 35 mm disc followed by the addition of onjisaponin B at the indicated concentrations for 24 h. The cells were then incubated with crystal violet for 10 min followed by a ddH_2_O wash. The stained cells image was captured by CCD digital camera Spot RT3™ under the Nikon ECLIPSE 80i microscope with 4× magnification. Cell viability was quantified by dissolving stained cells in 10% acetic acid (200 μL/well). The colorimetric reading of the solute mixture was then determined by spectrophotometer at OD 570 nm. The percentage of cell viability was calculated using the following formula: Cell viability (%) = Cells number_treated_/Cells number_DMSO control_ × 100. Data were obtained from three independent experiments. Cell viability was also measured by using Annexin V staining kit (BD Biosciences, San Jose, CA, USA). In brief, PC-12 cells with A53T mutant α-synuclein overexpression were treated with 50 μM of onjisaponin B. Cells were then harvested and analyzed by multiparametric flow cytometry using FITC-Annexin V and Propidium iodide staining (BD Biosciences, San Jose, CA, USA) according to the manufacturer’s instructions. Flow cytometry was then carried out using a FACSCalibur flow cytometer (BD Biosciences, San Jose, CA, USA). Data acquisition and analysis were performed with CellQuest (BD Biosciences, San Jose, CA, USA).

### 3.6. Western Blot

After drug treatments, cells were harvested and lysed in RIPA buffer from Cell Signaling Technologies Inc. (Beverly, MA, USA). Protein concentrations were measured by Bradford reagent (Bio-Rad, Hercules, CA, USA). The cell lysates were then resolved by SDS-PAGE. After electrophoresis, the proteins from SDS-PAGE were transferred to nitrocellulose membrane which was then blocked with 5% non-fat dried milk for 60 min. The membrane was then incubated with corresponding primary antibodies overnight at 4 °C. After that, the membrane was further incubated with HRP-conjugated secondary antibodies for 60 min. Finally, protein bands were visualized by using the ECL Western Blotting Detection Reagents (Invitrogen, Paisley, Scotland, UK). Band intensities were quantified by using the software ImageJ (ImageJ 1.46r; National Institutes of Health, Bethesda, MD, USA).

### 3.7. Real Time PCR Analysis

In brief, PC-12 cells transfected with EGFP-HDQ 74 were incubated with onjisaponin B for 16 h before RNA extraction. Total RNA was extracted from PC-12 cells by using FavorPrep™ Total RNA purification mini kit (Favorgen, Ping Tung, Taiwan). cDNA were synthesized by performing reverse transcription using SuperScript^®^ VILO™ Master Mix (Invitrogen, Grand Island, NY, USA). Real-time PCR were carried out on ViiA™ 7 Real Time PCR System (Applied Biosytems, Grand Island, NY, USA) using the FS Universal SYBR Green Master Rox (Roche, Indianapolis, IN, USA) according to manufacturer’s instructions. PCR was done by using primers 5′-ATG AAG GCC TTC GAG TCC CTC AAG TCC TTC-3′ and 5′-GGC GGC TGA GGA AGC TGA GAA-3′ as described in [66].

### 3.8. Removal of Mutant Huntingtin and Mutant α-Synuclein

PC-12 cells were transfected transiently with EGFP-HDQ 74 plasmid for 24 h using Lipofectamine Plus LTX reagent (Invitrogen, Grand Island, NY, USA) according to the manufacturer’s protocol. The transfected cells were then treated with onjisaponin B for 24 h. The removal of mutant huntingtin, EGFP-HDQ 74, was then quantitated by immunoblotting with antibody against GFP. To measure the clearance of α-synuclein in cellular model, the overexpression of mutant A53T α-synuclein was first induced by the addition of doxycycline (1 μg/mL) for 24 h [11] by using the doxycycline inducible PC-12 cell line transfected with A53T α-synuclein plasmid. The expression of mutant A53T α-synuclein was then switched off by removing doxycycline from medium. Cells were then incubated with *Radix Polygalae* extract or onjisaponin B for a further 24 h. The clearance of mutant α-synuclein was then measured by immunoblotting with antibody against myc-tag. Both the doxycycline inducible PC-12 cell line and A53T α-synuclein plasmid were generous gifts from C.B. KO (The Hong Kong Polytechnic University, Hong Kong, China).

### 3.9. Bimolecular Fluorescence Complementation (BiFC) Assay

GFP-N terminal-α-synuclein (GNS) and α-synuclein-GFP-C terminal (SGC) plasmids were kindly provided by Pamela J. McLean (Department of Neuroscience, Mayo Clinic Florida, Jacksonville, FL, USA). In brief, HeLa cells transfected with both GNS and SGC plasmids were incubated at 37 °C for 4 h. Then, the transfected cells were incubated with different concentrations of *Radix Polygalae* extract or onjisaponin B for a further 24 h at 30 °C [59]. Fluorescent signals upon complete GFP fluorophore reconstitution in cells were then detected by flow analysis (BD FACSAria III, San Jose, CA, USA).

### 3.10. Statistical Analysis

The results were expressed as means ± S.D. as indicated. The difference was considered statistically significant when the *p*-value was less than 0.05. Student’s *t*-test or one-way ANOVA analysis was used for comparison among different groups.

## 4. Conclusions

By using an image-based GFP-LC3 screening assay, our present study reports for the first time that onjisaponin B, isolated from the *Radix Polygalae*, is a potent compound for induction of autophagy, namely an autophagy inducer. The autophagy affect was confirmed by using wild type *Atg*7 and autophagy resistant *Atg*7-deficient mouse embryonic fibroblasts. Also, the results showed that onjisaponin B-mediated autophagy induction is dependent on the *Atg*7 and AMPK-mTOR signaling pathway. Moreover, both the ethanol extracts of *Radix Polygalae* and onjisaponin B derived from those extracts may play a protective role in neurodegenerative diseases by accelerating the removal of overexpressed mutant proteins such as A53T α-synuclein and huntingtin with 74 CAG repeats, as well as by reducing α-synuclein oligomerization in cells. Furthermore, although onjisaponin B is a novel autophagy enhancer with a potential neuroprotective role in the cellular model, the association between its autophagic activity and neuroprotective mechanism remains to be elucidated.
